# Benefits and harms of screening for and treatment of asymptomatic bacteriuria in pregnancy: a systematic review

**DOI:** 10.1186/s12884-016-1128-0

**Published:** 2016-11-02

**Authors:** Konstanze Angelescu, Barbara Nussbaumer-Streit, Wiebke Sieben, Fülöp Scheibler, Gerald Gartlehner

**Affiliations:** 1 Institute for Quality and Efficiency in Health Care (IQWiG), Cologne, Germany; 2 Department for Evidence-based Medicine and Clinical Epidemiology, Danube University Krems, Krems, Austria; 3 RTI-International, Chapel Hill, NC USA

**Keywords:** Mass Screening, Bacteriuria, Pregnancy, Benefit Assessment, Systematic Review

## Abstract

**Background:**

Most European and North American clinical practice guidelines recommend screening for asymptomatic bacteriuria (ASB) as a routine pregnancy test. Antibiotic treatment of ASB in pregnant women is supposed to reduce maternal upper urinary tract infections (upper UTIs) and preterm labour. However, most studies supporting the treatment of ASB were conducted in the 1950s to 1980s. Because of subsequent changes in treatment options for ASB and UTI, the applicability of findings from these studies has come into question. Our systematic review had three objectives: firstly, to assess the patient-relevant benefits and harms of screening for ASB versus no screening; secondly, to compare the benefits and harms of different screening strategies; and thirdly, in case no reliable evidence on the overarching screening question was identified, to determine the benefits and harms of treatment of ASB.

**Methods:**

We systematically searched several bibliographic databases, trial registries, and other sources (up to 02/2016) for randomised controlled trials (RCTs) and prospective non-randomised trials. Two authors independently reviewed abstracts and full-text articles and assessed the risk of bias of the studies included. As meta-analyses were not possible, we summarised the results qualitatively.

**Results:**

We did not identify any eligible studies that investigated the benefits and harms of screening for ASB versus no screening or that compared different screening strategies. We identified four RCTs comparing antibiotics with no treatment or placebo in 454 pregnant women with ASB. The results of 2 studies published in the 1960s showed a statistically significant reduction in rates of pyelonephritis (odds ratio [OR] = 0.21, 95 % confidence interval [CI] 0.07–0.59) and lower UTI (OR = 0.10, 95 % CI 0.03–0.35) in women treated with antibiotics. By contrast, event rates reported by a recent study were not statistically significantly different, neither regarding pyelonephritis (0 % vs. 2.2 %; OR = 0.37, CI 0.01–9.25, *p* = 0.515) nor regarding lower UTI during pregnancy (10 % vs. 18 %; Peto odds ratio [POR] = 0.53, CI 0.16–1.79, *p* = 0.357). Data were insufficient to determine the risk of harms. As three of the four studies were conducted several decades ago and have serious methodological shortcomings, the applicability of their findings to current health care settings is likely to be low. The recent high-quality RCT was stopped early due to a very low number of primary outcome events, a composite of preterm delivery and pyelonephritis. Therefore, the results did not show a benefit of treating ASB.

**Conclusions:**

To date, no reliable evidence supports routine screening for ASB in pregnant women.

**Electronic supplementary material:**

The online version of this article (doi:10.1186/s12884-016-1128-0) contains supplementary material, which is available to authorized users.

## Background

An amount of ≥ 10^5^ bacteria per ml of freshly voided urine and the absence of typical symptoms of urinary tract infection (UTI) is referred to as asymptomatic bacteriuria (ASB) [1, 2]. The reported prevalence rates in pregnancy range from 2 to 15 % [3–6]. Generally a benign condition in most adults, in pregnant women ASB has been associated with an increased risk of complications, especially upper UTIs (pyelonephritis) and preterm birth [7–9].

While pyelonephritis normally requires hospitalisation and sometimes leads to severe complications such as sepsis and respiratory problems [10], preterm birth is the main contributor to infant morbidity and mortality. Most clinical practice guidelines therefore recommend screening for and antibiotic treatment of ASB in pregnancy [1, 2, 11, 12]. In most health care systems a screening programme for ASB in pregnancy has long been part of routine maternal care [13].

These recommendations are based on data published in the 1960s to 1980s and summarised in an update of a Cochrane Review on the antibiotic treatment of ASB in August 2015 [14]. Our systematic review had an extended scope comprising three objectives: firstly, to assess the patient-relevant benefits and harms of screening for ASB versus no screening; secondly, to compare the benefits and harms of different screening strategies; and thirdly, in case no reliable evidence on the overarching screening question was identified, to determine the benefits and harms of treatment of ASB.

This systematic review is an update of a health technology assessment report of the benefits and harms of screening for ASB in pregnancy conducted by the German Institute for Quality and Efficiency in Health Care (Institut für Qualität und Wirtschaftlichkeit im Gesundheitswesen, IQWiG).

## Methods

### Protocol and methodological approach

The full (German-language) protocol and report [15], as well as an English-language executive summary, are available on the Institute’s website [16]. Both the preliminary protocol and the preliminary report underwent public commenting procedures. IQWiG’s responsibilities and methodological approach are described in its methods paper [15]. Only previously published studies were used, so there was no requirement for ethical review and consent.

### Search strategy and study selection

Primary studies and secondary publications were searched for in MEDLINE (1946 to January 2016) and EMBASE (1974 to January 2016) via Ovid, and in the Cochrane Central Register of Controlled Trials (January 2016). The Cochrane Database of Systematic Reviews, the Database of Abstracts of Reviews of Effects, and the Health Technology Assessment Database were screened to identify systematic reviews. Reference lists of retrieved systematic reviews were searched by hand. In addition, web-based clinical trial registries were screened (ClinicalTrials.gov, International Clinical Trials Registry Platform Search Portal, and the EU Clinical Trials Register). The search strategy included bibliographic index terms on bacteriuria and pregnancy. The complete search strategy, which was developed by one information specialist and checked by another, is presented in Additional file 1. We also screened publications cited in comments addressed to the Federal Joint Committee, the decision-making body in the German statutory healthcare system and IQWiG’s main commissioning body. In addition, persons and parties who had submitted written comments on the preliminary report were asked to provide any additional relevant studies. Two reviewers independently screened titles and abstracts of retrieved citations to identify potentially eligible primary and secondary publications. The full texts of these articles were obtained and independently evaluated by the same two reviewers applying the full set of inclusion and exclusion criteria. All documents retrieved from non-bibliographic sources were also screened for eligibility or relevant information on studies. Disagreements were resolved by consensus.

### Eligibility criteria

#### Study characteristics

We included randomised controlled trials (RCTs). If insufficient evidence was available from RCTs, we also planned to include non-randomised interventional prospective trials (referred to as controlled clinical trials: CCTs).

The eligibility criteria for the population, study and control interventions, and outcomes are presented in Table 1.

**Table 1:** Eligibility criteria

	Questions 1 and 2 (ASB screening)	Question 3 (ASB treatment)
Population	• Pregnant women taking part in routine maternal care • Without symptoms of UTI • With unknown ASB status	• Pregnant women with ASB detected in screening
Study intervention	• Any ASB screening strategy followed by treatment, if necessary	• Any treatment for ASB
Control intervention	• No ASB screening, but treatment if symptoms of UTI occur (question 1) • Any other ASB screening strategy followed by treatment, if necessary (question 2)	• No treatment or placebo
Patient-relevant outcomes	• Pyelonephritis • UTI • Symptoms linked directly or indirectly to UTI (e. g. headache or visual impairment as symptoms of pre-eclampsia, fever) • Infant morbidity (e. g. respiratory distress syndrome, sepsis, cerebral haemorrhage, necrotising enterocolitis) • Perinatal mortality • Early preterm birth (< 32 weeks of gestation) • Very low birth weight (< 1500 g) • Health-related quality of life and psychosocial functioning • Any adverse event	

*ASB* asymptomatic bacteriuria, *UTI* urinary tract infection

We searched for studies investigating at least one predefined patient-relevant outcome. In this context, the term ‘patient-relevant’ refers to how a person (in this case, a mother or child) feels, functions, or survives [17]. In addition, we planned to analyse data on the following additional predefined non-patient-relevant outcomes if they were reported in the studies included: preterm birth > 32 − < 37 weeks of gestation, birth weight 1500 − < 2500 g, and pre-eclampsia with unknown symptom status.

### Document characteristics

We included both published and unpublished studies if a full-text document (e. g. journal article or clinical study report) was available. We did not apply language or publication date restrictions. We excluded multiple publications not providing additional relevant information.

### Data extraction

The individual steps of the data extraction and risk of bias assessment were conducted by one author and checked by another; disagreements were resolved by consensus. We extracted details of the studies using standardised tables developed and routinely used by IQWiG.

We extracted information from each included study on:

1. Study characteristics, including study design, length of follow-up, sample size, location, and period in which the study was conducted.
2. Characteristics of the study participants, including age, parity, present diabetes mellitus, history of UTI, sociodemographic data, and dropout rate.
3. Characteristics of interventions, including treatment regimen and adjunct treatments.
4. Inclusion and exclusion criteria, including method of urine collection, diagnostic procedure(s), and cut-offs used to identify study participants.
5. Risk-of-bias items (see below).

### Assessment of risk of bias

We assessed the risk of bias for individual studies, as well as for each outcome, and rated these risks as “high” or “low”. In individual studies the risk of bias was assessed by determining the adequacy of the following quality criteria: generation of random allocation sequence, allocation concealment, blinding of participants and investigators, and selective outcome reporting. As no CCTs were identified, no further details of the respective risk-of-bias assessment planned are provided here. If the risk of bias on the study level was rated as “high”, the risk of bias on the outcome level was generally also regarded as “high”. The risk of bias for each outcome was assessed by determining the adequacy of the following quality criteria: blinding of outcome assessors, application of the intention-to-treat (ITT) principle, and selective outcome reporting.

### Data analysis

We performed a synthesis and analysis of information by means of the methods described below and presented a summarising evaluation. Results of outcomes retrieved from individual studies were described comparatively. For the statistical analysis, we planned to primarily use results from the ITT analysis as reported. If not provided, we calculated the required estimates of location and dispersion. We reported the treatment effects as ORs (including 95 % CIs) for binary data and planned to report mean differences (including 95 % CIs) for continuous data. We planned to assess potential heterogeneity of effect sizes by means of the I^2^ statistic and a statistical heterogeneity test [18]; if relevant heterogeneity was shown (p < 0.2), we planned to calculate pooled estimates only in justified exceptional cases. As meta-analyses were not feasible, we assessed the results of the individual studies. We performed sensitivity analyses to explore the potential impact of missing data.

## Results

As we did not identify any eligible studies investigating the benefits and harms of screening for ASB versus no screening, or the advantages and disadvantages of different screening strategies, our results focus on the treatment of ASB.

### Literature search

**Fig. 1:**
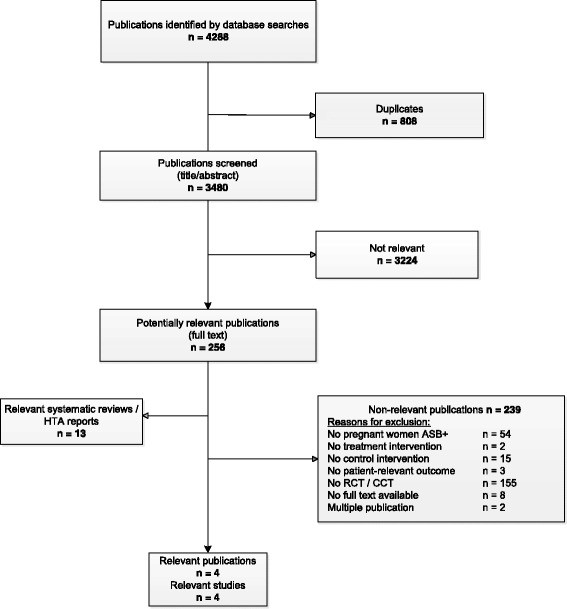
Flowchart of study selection

We identified four eligible studies out of 4288 references retrieved from bibliographic databases (Fig. 1): Elder et al. 1966 [19], Mulla 1960 [20], Kazemier et al. 2015 [21], and Williams et al. 1969 [22]. All four studies were RCTs assessing the treatment of ASB, one of which was embedded in a multicentre cohort screening study (Kazemier 2015).

### Study characteristics

Table 2 presents the main characteristics of the four studies included. Three were published in the 1960s. One study enrolled patients from 2011 to 2013 and the results were published in 2015. The four studies investigated the effect of antibiotic treatment in 454 pregnant women with ASB. The total number of randomised participants is unknown, due to a lack of data in one study (Williams et al.). Only one study (Kazemier et al.) provided information on patient characteristics.

**Table 2:** Study characteristics

Comparison study	Study design	Participants randomised (intervention / control)	Treatment regimen	Location / Setting / recruitment period	Length of follow-up	Urine collection / methods used for diagnosis	Inclusion and exclusion criteria
Sulphasymazine vs. placebo							
Elder [19]	RCT, double-blind, parallel	106 (54 / 52)	Sulphasymazine 0.5 g/d until birth or onset of pyelonephritis; if bacteriuria persisted medication was changed to nitrofurantoin or tetracycline, dosage not stated	USA / outpatient maternal care / 06 / 1965 – 03 / 1966	Until immediately after birth^a^	Clean voided / UC	I: pregnant; same species of bacteria in first 3 uncontaminated^b^ specimens, ≥ 10^4^ /ml in one and ≥ 10^5^/ml in 2 E: > 32th week of gestation at first examination
Sulphadimethoxine vs. no treatment							
Mulla [20]	RCT, blinding not stated	100 (50 / 50)	Sulphadimethoxine 2 x 250 mg/d 7 days; if bacteriuria persisted treatment was repeated	USA / not stated / not stated	Until immediately after birth^a^	Catheter (not specified) / UC and “stained smear”^c^ not further specified	I: 30th – 32th week of gestation; bacteriuria (not specified) E: not stated
Sulphadimidine vs. no treatment							
Williams [22]	RCT, blinding not stated	Not stated (85 / 78)^d^	Sulphadimidine 3 x 1 g 7 days; if bacteriuria persisted until 2 to 3 weeks after finishing primary treatment, then nitrofurantoin 2 x 100 mg/d for 7 days if still persisting ampicillin 3 x 250 mg for 7 days	GB / maternal care / 1967	10 days post partem	Voided midstream / UC	I: < 30th week of gestation at recruitment; > 10^5^ g-negative bacteria /ml in ≥ 2 consecutive specimens E: not stated
Nitrofurantoin vs. placebo							
Kazemier [21]	Double-blind, placebo-controlled RCT, embedded in a multicentre cohort study	85 (40 / 45)	Nitrofurantoin 100 mg 2x/d 5 days, self-administered if follow-up culture positive one week after end of treatment, 1x repeated (masked) medication at the same dose and schedule	NL / hospitals and ultrasound centres / 10 / 2011 – 6 / 2013^e^	Until 08 / 2014	Not stated / dip slide with two culture media	I: women ≥ 18 years with a singleton pregnancy between 16 and 22 weeks; without symptoms of UTI; ≥ 1x10^5^ CFU /ml of a single microorganism or when 2 different colony types were present but 1 with ≥ 1x10^5^ CFU / ml E: history of preterm delivery < 34 weeks; warning signs of an imminent preterm delivery; fetal congenital malformations; antibiotic use within 2 weeks of screening; known glucose-6-phosphate dehydrogenase deficiency; hypersensitivity to nitrofurantoin; risk factors for complicated UTI

*CFU* colony forming units, *GB* Great Britain, *I* inclusion criteria, *E* exclusion criteria, *g* gram, *ml* millilitre, *NL* Netherlands, *RCT* randomised controlled trial, *UC* urinary culture, *USA* United States of America, *UTI* urinary tract infection

^a^Exact length of follow-up not stated, but outcomes were assessed that occurred immediately after birth

^b^Contamination was defined as a specimen with “large numbers of organisms that were likely to be of vaginal origin”

^c^The diagnostic strategy to identify the study population consisted of two different diagnostic tests. No details were reported on the specific catheter, the number of positive test results required, the cut-offs used, or the diagnostic algorithm (i.e., whether both tests were used as a combination and, if so, how the test results were combined to diagnose ASB)

^d^Of originally 211 pregnant women with gram-negative asymptomatic bacteriuria, a subgroup of participants restricted to those with coliform bacteria was analysed in the relevant trial

^e^Refers to the entire cohort study

Elder et al. investigated the effect of sulfasymazine on multiple laboratory parameters and selected adverse events in pregnant women with and without ASB. Pyelonephritis cases were also mentioned but event rates were missing.

Kazemier et al., a multicentre prospective cohort study with an embedded RCT conducted in the Netherlands, included a cohort of low-risk pregnant women screened for ASB using the dip slide method. Consenting ASB-positive women were randomised to nitrofurantoin or placebo. The aim of the RCT was to evaluate the effect of nitrofurantoin on a composite primary outcome, preterm delivery and pyelonephritis, as well as on a number of secondary maternal and neonatal outcomes. The study was stopped early after the planned interim analysis when data were available for 30 % of the planned sample, as pyelonephritis events were much less frequent than expected in both the treatment and control groups.

Mulla provided only a vague description of the study characteristics (see Table 2). In particular, details were missing on the diagnostic strategy used. No information was provided on the specific catheter, the number of positive test results, the cut-offs, and the diagnostic algorithm (i.e., whether both tests were used as a combination and, if so, how the test results were combined to diagnose ASB).

Williams et al. reported the results of two consecutive trials, one of which is relevant for this systematic review. Prior to the relevant trial, the majority of participants had taken part in a preceding trial where they had been hospitalised for 24 h, while fluid intake had been restricted to a minimum.

### Risk of bias

In the three studies from the 1960s the risk of bias was high for nearly all items considered, while the recent trial (Kazemier et al.) had a low risk of bias on the study and outcome level (see Table 3).

**Table 3:** Risk of bias of included trials

Study	Randomisation appropriate	Allocation concealment appropriate	Blinding patient / investigator / outcome assessor	Selective reporting improbable	Absence of other factors potentially causing bias	ITT analysis appropriate	Risk of bias (study level)
Elder [19]	unclear	unclear	yes / unclear / unclear	unclear^a, b^	no^c^	no^d^	high
Mulla [20]	unclear	unclear	no / no / unclear	no^a, e^	no^c, f^	unclear	high
Williams [22]	unclear	unclear	no / no / unclear	no^a, g^	no^c^	no^h^	high
Kazemier [21]	yes	yes	yes / yes / yes	yes	yes	yes	low

^a^Sample size planning, predefinition of study outcomes and their analysis not reported

^b^The outcome “kernicterus” was reported together with other adverse outcomes, some of which were reported for only one study group

^c^Patient flow unclear; unclear whether information on inclusion and exclusion criteria was complete

^d^Some participants were excluded from the analysis; information on study discontinuations was insufficient

^e^The outcome “preterm labour” was reported only for the control group; one outcome usually reported in association with preterm labour, preterm birth, was not reported here

^f^The outcome “cystopyelitis” was not defined and it was therefore unclear whether upper and / or lower UTI were included

^g^Results of one outcome not relevant to this assessment were reported incompletely

^h^Some participants were excluded from the analysis; the reasons were not stated

### Effects of interventions

#### Pyelonephritis and lower UTI

Williams et al. reported results on pyelonephritis, Mulla on lower UTI, and Kazemier et al. on both outcomes (Table 4): In Williams et al. and Mulla, respectively, antibiotic treatment statistically significantly reduced the incidence of pyelonephritis (6 % vs. 23 %; OR = 0.21, 95 % CI 0.07–0.59, *p* = 0.002) and of lower UTI (6 % vs. 40 %; OR = 0.10, 95 % CI 0.03–0.35, *p* < 0.001). By contrast, event rates reported by Kazemier et al. were not statistically significantly different for pyelonephritis (0 % vs. 2.2 %; OR = 0.37, CI 0.01–9.25, *p* = 0.515) and lower UTI treated with antibiotics during pregnancy (10 % vs. 18 %; POR = 0.53, CI 0.16–1.79, *p* = 0.357) or during a 6-week postpartum period (see Table 4). Because of substantially different study periods (1960s vs. 2010s), pooling of data was not feasible.

**Table 4:** Results

Outcome measure	Study	Treatment group		Control group		Difference between groups
Specification		*N*	Events (%)	*N*	Events (%)	OR [95 % CI]; *p*-value
Pyelonephritis						
	Kazemier [21]	40	0 (0)	45	1 (2.2)	0.37^a^ [0.01; 9.25]^a^; 0.515^b^
	Williams [22]	85^c^	5 (6)	78^c^	18 (23)	0.21^a^ [0.07–0.59]^a^; 0.002^b^
Lower UTI						
Treated with AB during pregnancy	Kazemier [21]	40	4 (10)	45	8 (18)	POR 0.53^a^ [0.16; 1.79]^a^; 0.357^b^
Recurrent UTI treated with AB during pregnancy	Kazemier [21]	40	0 (0)	45	1 (2.2)	0.37^a^ [0.01; 9.25]^a^; 0.515^b^
Treated with AB postpartum (within 6 weeks)	Kazemier [21]	40	3 (7.5)	45	1 (2.2)	POR 3.20^a^ [0.43; 23.63]^a^; 0.296^b^
Pre- and post-partal^d^	Mulla [20]	50	3 (6)	50	20 (40)	0.10^a^ [0.03–0.35]^a^; < 0.001^b^
Preterm birth						
< 37 weeks^e^	Kazemier [21]	40	2^f^ (5)	45	2 (4.4)	POR 1.13^a^ [0.15; 8.35]^a^; 0.975^b^
< 32 weeks	Kazemier [21]	40	1 (2.5)	45	0 (0)	3.46^a^ [0.14; 87.26]^a^; 0.357^b^
Infant morbidity						
Kernicterus	Elder [19]	54	0^g^	52	0^g^	n. a.
Composite severe morbidity^h^	Kazemier [21]	40	0 (0)	45	2 (4.4)	0.21^a^ [0.01; 4.61]^a^; 0.220^b^
Admission to NICU	Kazemier [21]	40	2 (5)	45	0 (0)	5.91^a^ [0.28; 126.85]^a^; 0.169^b^
Neonatal sepsis confirmed with culture	Kazemier [21]	40	0 (0)	45	2 (4.4)	0.21^a^ [0.01; 4.61]^a^; 0.220^b^
Congenital abnormalities	Kazemier [21]	40	0 (0)	45	1 (2.2)	0.37^a^ [0.01; 9.25]^a^; 0.515^b^
Infant mortality						
Perinatal death	Kazemier [21]	40	1 (2.5)	45	0 (0)	3.46^a^ [0.14; 87.26]^a^; 0.357^b^
Adverse events						
Vomiting	Elder [19]	54	1	52	0	n. a.
Rashes, pruritus	Elder [19]	54	0^f^	52	0^f^	n. a.
Photosensitivity	Elder [19]	54	0^f^	52	0^f^	n. a.
Discontinuations due to adverse events	Mulla [20]	50	0	50	0	n. a.
Pre-eclampsia^e^	Kazemier [21]	40	2 (5)	45	1 (2.2)	POR 2.24^a^ [0.23; 22.22]^a^; 0.596^b^
HELLP syndrome	Kazemier [21]	40	2 (5)	45	0 (0)	5.91^a^ [0.28; 126.85]^a^; 0.169^b^
Kidney stones, cholestasis	Kazemier [21]	40	0 (0)	45	0 (0)	RD 0 [-9,4; 10,5]
Thrombo-embolic events	Kazemier [21]	40	0 (0)	45	0 (0)	RD 0 [-9,4; 10,5]
Endometritis (within 6 weeks of delivery)	Kazemier [21]	40	0 (0)	45	0 (0)	RD 0 [-9,4; 10,5]
Mastitis (within 6 weeks of delivery)	Kazemier [21]	40	1 (2.5)	45	1 (2.2)	POR 1.13^a^ [0.07; 18.41]^a^; 0.997^b^

*AB* antibiotics, *CI* confidence interval, *CSZ* convexity, symmetry, z score, *HELLP* haemolysis, elevated liver enzymes, low platelet count syndrome, *n. a* not available, *NICU* Neonatal Intensive Care Unit, *OR* odds ratio, *POR* Peto odds ratio, *RD* risk difference, *UTI* urinary tract infection

^a^IQWiG’s own calculation

^b^IQWiG’s own calculation, unconditioned exact test (CSZ method as described in [25])

^c^Number of participants analysed; number of randomised participants not stated

^d^The outcome was named either cystopyelitis or symptomatic UTI; neither term was defined. It was therefore unclear which stage of UTI the reported outcome represented. Following a conservative approach, we classified the outcome as lower UTI. However, it is possible that cases of upper UTI were also included

^e^Considered a non-patient-relevant outcome

^f^One event is also included in preterm births < 32 weeks

^g^It is unclear whether the reported event rate relates to both study groups; alternatively, the event rate may relate solely to the treatment group or to any pregnant participant with or without bacteriuria

^h^Respiratory distress syndrome, necrotizing enterocolitis, intraventricular haemorrhage, bronchopulmonary disease, sepsis

In the analysis of pyelonephritis rates in Williams et al. the proportion of excluded participants was high (22.7 %). We therefore performed a sensitivity analysis to assess the impact of missing data, assuming that all participants (211 instead of the 163 actually analysed) were included, and both the treatment and control group were of equal size (Table 5). Under the assumption that no events had occurred in excluded participants, the effect remained statistically significant. Assuming that additional events had occurred only in excluded women of the treatment group, the effect remained statistically significant until at least four additional events occurred in this group (with the events in the control group remaining unchanged).

**Table 5:** Sensitivity analysis

Outcome measure	Treatment group		Control group		Difference between groups
Study	*N*	Events (%)	*N*	Events (%)	OR [95 % CI]; *p*-value
Pyelonephritis					
Williams [22] Sensitivity analysis I	106^a^	5 (5)	105^a^	18 (17)	0.24^b^ [0.19–1.05]^b^; 0.004^c^
Williams [22] Sensitivity analysis II	106^a^	9 (8)^d^	105^a^	18 (17)	0.45^b^ [0.19–1.05]^b^; 0.066^c^

*CI* confidence interval, *CSZ* convexity, symmetry, z score, *OR* odds ratio

^a^IQWiG’s own calculation: all participants included (211 instead of the 163 actually analysed); both the treatment and control group were of equal size

^b^IQWiG’s own calculation

^c^IQWiG’s own calculation, unconditioned exact test (CSZ method as described in [25])

^d^The level of significance would not be missed until at least four additional events occurred in the treatment group while no additional events occurred in the control group (assumption)

### Preterm birth

One study (Kazemier et al.) provided data on preterm birth. Preterm birth rates (<37 weeks of gestation) were low in both groups and there was no statistically significant difference (5.0 % vs. 4.4 %, Peto odds ratio [POR] = 1.13, CI 0.15–8.35, *p* = 0.975). Only one preterm birth event considered patient-relevant, i.e. preterm birth < 32 weeks, occurred in the interventional arm (see Table 4).

### Infant morbidity

Only one study (Kazemier et al.) contained analyses of infant outcomes. Event rates, in general, were low and did not reveal any statistically significant difference between study groups (see Table 4).

### Perinatal mortality

One study reported data on perinatal mortality (Kazemier et al.). The difference was not statistically significant, as there was only one case in the interventional arm (see Table 4).

### Adverse events

The available data did not allow conclusions to be drawn on adverse events, as in one study (Elder et al.) the event rate in the control group was not clearly stated, while no events (Mulla) or very few (Kazemier et al.) occurred in the other two studies (see Table 4). We therefore could not determine the risk of adverse events under antibiotic treatment, placebo or no treatment.

### Other outcomes

None of the studies reported data on further predefined patient-relevant outcomes such as symptoms linked directly or indirectly to UTI, birth weight < 1500 g, health-related quality of life, and psychosocial functioning. One study reported data on the predefined non-patient-relevant outcome “pre-eclampsia” (Kazemier et al.) without revealing any statistically significant difference between study groups (5 % vs. 2.2 %, POR = 2.24, CI 0.23–22.22, *p* = 0.596).

## Discussion

### Summary of evidence

No studies were identified on the primary research question, the benefit of screening for ASB versus no screening.

Four RCTs, of which three had a high risk of bias, included data on 454 patients and provided limited information on the benefits and harms of antibiotic treatment for women with ASB. Data collected more than 50 years ago indicate a reduction in the risk of UTIs and pyelonephritis for women receiving antibiotic treatment, whereas recent results of a high-quality RCT failed to show any statistically significant difference.

The inconsistent results and the fact that three studies were conducted more than 50 years earlier than the most recent study raise the question of the applicability of their findings.

### Applicability

As the screening tests used in Mulla were not described, it is not known how women who benefited from treatment were identified. In Williams et al., the above-mentioned interventions preceding the relevant trial caused urinary concentration, an increase in the risk of nosocomial infections, and a delay in treatment. The setting created differs considerably from current routine maternity care and may interact with the UTI outcomes described below [23, 24]. This means that, although both trials suggest a preventive effect of treatment of ASB with regard to upper and lower UTI, the results do not allow conclusions to be drawn about today’s pregnant women in current health care settings. This is also reflected in the results of the study by Kazemier et al. Consequently, the benefit of treatment of ASB to prevent upper and lower UTI is regarded as not proven.

Besides, further aspects challenge the applicability of the three studies from the 1960s. None of them contained details on age, parity, previous and concomitant diseases, and sociodemographic data of the study population. It was thus difficult to judge comparability with today’s pregnant women. Factors that have undergone considerable changes since the 1960s and may influence the effects of treatment of ASB include the content and scope of routine maternal care services, the general health status and demographic characteristics of pregnant women, as well as the further development of diagnostic procedures. In summary, these factors have resulted in a lower baseline risk of pyelonephritis [21].

The study medication in the three studies from the 1960s consisted primarily of sulphonamides, while only the recent Dutch study used nitrofurantoin, a drug currently used as a first-line treatment option for ASB.

### Comparison with previous research

Due to differing inclusion and exclusion criteria, the above-mentioned Cochrane Review [14] included far more studies than our review (14 randomised and quasi-randomised trials). The Cochrane Review considered the primary outcomes pyelonephritis, low birth weight, and preterm birth. A meta-analysis of 11 of the 14 studies showed a statistically significant decline in pyelonephritis rates under antibiotic treatment compared with no treatment (RR [relative risk] = 0.23, 95 % CI 0.13–0.41, *p* < 0.001).

The authors of the Cochrane Review conclude that antibiotics are effective in preventing pyelonephritis, but also state that the methodological shortcomings of the trials included affect the strength of their conclusions.

However, our review comprises results of the first trial in this field that has been conducted since the 1980s, which leads to considerable changes in the judgement of the benefits of screening and treating ASB.

### Strengths and limitations (study and review level)

As we anticipated publications of varying quality and age, the inclusion and exclusion criteria for the study population were applied rather strictly in order to obtain sufficiently conclusive data. We placed particular emphasis on the requirement that the population should consist solely of asymptomatic women. When we could not rule out that symptomatic women were included, the study was not considered. This resulted in a study pool of only four eligible studies.

The existing evidence derived from RCTs refers only to the treatment of ASB. RCTs investigating the overarching question, that is, whether a screening programme for detection of ASB is beneficial, have still not been conducted. However, the results of the Dutch ASB cohort study show that, with access to high-quality health care and generally good maternal health status, a routine ASB screen-and-treat policy may not result in a considerable gain in health outcomes.

## Conclusions

### Interpretation of results

To date, no RCTs are available that assess the benefits and harms of screening for ASB. The available evidence is limited to four treatment trials: three with serious methodological shortcomings and questionable applicability to current medical practice and one low-risk-of-bias trial that was stopped due to a very low number of pyelonephritis events in both the treatment and control group. Consequently, no conclusions can be drawn on whether the benefits of screening for ASB outweigh the potential harms. However, no reliable evidence supports routine screening for ASB in pregnant women.

### Implications for future research

Due to the low number of women randomised (*n* = 85), the randomised part of the study by Kazemier et al. provides little additional information on the question as to whether treatment of ASB is beneficial. However, the low absolute risk of pyelonephritis in low-risk pregnancies questions current practice. Taking this low risk into account, future trials have to be planned on a larger scale to achieve sufficient statistical power to either confirm or adjust current recommendations on screening for and treatment of ASB. However, as long as there are no new studies indicating the need to screen and treat ASB in pregnancy, screening is not supported by clinical evidence.

## Additional file

Additional file 1: Search strategy. The file contains the full description of the search strategy. (PDF 18 kb)

## Abbreviations

ASB+: Positive for asymptomatic bacteriuria
CCT: Controlled clinical trial
HTA: Health technology assessment
RCT: Randomised controlled trial
