# Supplementary material for: Benefits and harms of screening for and treatment of asymptomatic bacteriuria in pregnancy: a systematic review
Source: BMC Pregnancy Childbirth. 2016 Nov 2;16:336. doi: 10.1186/s12884-016-1128-0 (PMC5093995; doi:10.1186/s12884-016-1128-0)
Supplement: Additional file 1: — Search strategy. The file contains the full description of the search strategy. (PDF 18 kb) [file 12884_2016_1128_MOESM1_ESM.pdf]

Additional file 1:

Search strategy

1. EMBASE

Search interface: Ovid

Embase 1974 to 2016 January 29

The following search filters were applied:

RCT: Wong [1] – High sensitivity strategy

Systematic review: Wong [1] – Strategy minimizing difference between sensitivity and specificity

#	**Searches**
1	*urinary tract infection/
2	exp Bacteriuria/
3	exp Asymptomatic Bacteriuria/
4	exp Streptococcus/
5	bacteriuri*.ti,ab.
6	(urinary* adj3 infection*).ti,ab.
7	streptococc*.ti,ab.
8	or/1-7
9	exp Pregnancy Complications/
10	exp Pregnancy/
11	pregnan*.ti,ab.
12	or/9-11
13	8 and 12
14	random*.tw.
15	clinical trial*.mp.
16	exp health care quality/
17	or/14-16
18	13 and 17
19	meta analysis*.mp.
20	search*.tw.
21	review.pt.
22	or/19-21
23	13 and 22
24	18 or 23
25	24 not medline*.cr.

2. MEDLINE

Search interface: Ovid

- Ovid MEDLINE(R) In-Process & Other Non-Indexed Citations January 29, 2016
- Ovid MEDLINE(R) 1946 to January Week 3 2016
- Ovid MEDLINE(R) Daily Update January 29, 2016

The following search filters were applied:

RCT: Lefebvre [2] – Cochrane Highly Sensitive Search Strategy for identifying randomized trials in MEDLINE: sensitivity-maximizing version (2008 revision)

Systematic review: Wong [1] – High specifity strategy

#	**Searches**
1	Bacteriuria/
2	*Urinary Tract Infections/
3	Streptococcal Infections/
4	bacteriuri*.ti,ab.
5	(urinary* adj3 infection*).ti,ab.
6	streptococc*.ti,ab.
7	or/1-6
8	exp Pregnancy/
9	pregnan*.ti,ab.
10	or/8-9
11	7 and 10
12	randomized controlled trial.pt.
13	controlled clinical trial.pt.
14	(randomized or placebo or randomly or trial or groups).ab.
15	drug therapy.fs.
16	or/12-15
17	(animals not (humans and animals)).sh.
18	16 not 17
19	11 and 18
20	cochrane database of systematic reviews.jn.
21	(search or MEDLINE or systematic review).tw.
22	meta analysis.pt.
23	or/20-22
24	11 and 23
25	19 or 24

3. PubMed

Search interface: NLM

- PubMed - as supplied by publisher
- PubMed - in process
- PubMed – OLDMEDLINE
- PubMed – pubmednotmedline

Search	Query
#1	Search bacteriuri*[tiab]
#2	Search (urinary*[tiab] AND infection*[tiab]))
#3	Search streptococc*[tiab]
#4	Search (#1 OR #2 OR #3)
#5	Search pregnan*[tiab]
#6	Search (#4 AND #5)
#7	Search (#6 not medline[sb])
#8	Search (clinical trial*[tiab] or random*[tiab] or placebo[tiab] or trial[ti])
#9	Search (#7 AND #8)
#10	Search (search[tiab] or meta analysis[tiab] or MEDLINE[tiab] or systematic review[tiab])
#11	Search (#7 AND #10)
#12	Search (#9 OR #11)

4. The Cochrane Library

Search interface: Wiley

- Cochrane Database of Systematic Reviews : Issue 1 of 12, January 2016
- Cochrane Central Register of Controlled Trials : Issue 1 of 12, January 2016
- Database of Abstracts of Reviews of Effect : Issue 2 of 4, April 2015
- Health Technology Assessment Database : Issue 1 of 4, January 2016

ID	Search
#1	MeSH descriptor: [Bacteriuria] this term only
#2	MeSH descriptor: [Urinary Tract Infections] this term only
#3	MeSH descriptor: [Streptococcal Infections] this term only
#4	#1 or #2 or #3
#5	bacteriuri*:ti,ab
#6	bacteriuri*
#7	(urinary* near/3 infection*):ti,ab

ID	Search
#8	urinary* near/3 infection*
#9	streptococc*:ti,ab
#10	streptococc*
#11	#4 or #5 or #7 or #9
#12	#4 or #6 or #8 or #10
#13	MeSH descriptor: [Pregnancy] explode all trees
#14	pregnan*:ti,ab
#15	pregnan*
#16	#13 or #14
#17	#13 or #15
#18	#11 and #16 in Cochrane Reviews (Reviews and Protocols) and Trials
#19	#12 and #17 in Other Reviews and Technology Assessments

References

1. Wong SSL, Wilczynski NL, Haynes RB. Comparison of top-performing search strategies for detecting clinically sound treatment studies and systematic reviews in MEDLINE and EMBASE. J Med Libr Assoc. 2006;94(4):451-5.
2. Lefebvre C, Manheimer E, Glanville J. Searching for studies. In: Higgins JPT, Green S, editors. Cochrane handbook for systematic reviews of interventions. New York: Wiley; 2008. p. 95-150.
